# Deuterium-depleted water stimulates GLUT4 translocation in the presence of insulin, which leads to decreased blood glucose concentration

**DOI:** 10.1007/s11010-021-04231-0

**Published:** 2021-09-12

**Authors:** Miklós Molnár, Katalin Horváth, Tamás Dankó, Ildikó Somlyai, Beáta Zs. Kovács, Gábor Somlyai

**Affiliations:** 1 Institute of Pathophysiology, Faculty of Medicine, Semmelweis University, Budapest, Hungary; 2 HYD LLC for Cancer Research and Drug Development, Villányi út 97, 1118 Budapest, Hungary

**Keywords:** Deuterium (D), Deuterium-depleted water (DDW), Deuterium depletion, Diabetes mellitus (DM), GLUT-4 translocation

## Abstract

Deuterium (D) is a stable isotope of hydrogen (H) with a mass number of 2. It is present in natural waters in the form of HDO, at a concentration of 16.8 mmol/L, equivalent to 150 ppm. In a phase II clinical study, deuterium depletion reduced fasting glucose concentration and insulin resistance. In this study, we tested the effect of subnormal D-concentration on glucose metabolism in a streptozotocin (STZ)-induced diabetic rat model. Animals were randomly distributed into nine groups to test the effect of D_2_O (in a range of 25–150 ppm) on glucose metabolism in diabetic animals with or without insulin treatment. Serum glucose, fructose amine-, HbA1c, insulin and urine glucose levels were monitored, respectively. After the 8-week treatment, membrane-associated GLUT4 fractions from the soleus muscle were estimated by Western blot technique. Our results indicate that, in the presence of insulin, deuterium depletion markedly reduced serum levels of glucose, -fructose amine, and –HbA1c, in a dose-dependent manner. The optimal concentration of deuterium was between 125 and 140 ppm. After a 4-week period of deuterium depletion, the highest membrane-associated GLUT4 content was detected at 125 ppm. These data suggest that deuterium depletion dose-dependently enhances the effect of insulin on GLUT4 translocation and potentiates glucose uptake in diabetic rats, which explains the lower serum glucose, -fructose amine, and –HbA1c concentrations. Based on our experimental data, deuterium-depleted water could be used to treat patients with metabolic syndrome (MS) by increasing insulin sensitivity. These experiments indicate that naturally occurring deuterium has an impact on metabolic regulations.

## Introduction

Deuterium (D), a naturally occurring stable isotope of hydrogen, is present in natural surface waters predominantly in the form of HDO and at a concentration of approximately 16.8 mmol/L. The two isotopes, hydrogen (^1^H) and deuterium (^2^H) have the largest mass difference among stable isotopes of the same element, resulting in significant differences in both chemical and physical properties [1–3].

The effect of D at elevated concentrations has been investigated thoroughly in various biological systems [4, 5], but these studies ignored the significance of natural D-concentrations.

In nature, the deuterium to hydrogen (D/H) ratio is approx. 1:6600; the natural abundance of D is about 150 ppm (0.015 atom%) [2]. Global concentration of D ranges between 120 and 160 ppm depending on the site of sampling [6, 7] and the available data suggest that D/H ratio is not constant in living organisms, either [8].

Deuterium in the human plasma is abundant with concentrations reaching 12–14 mmol/L, in comparison with the 2.24–2.74 mmol/L concentration of calcium, 0.75–1.2 mmol/L concentration of magnesium, 5.0–5.1 mmol/L value of potassium and 3.3–6.1 mmol/L circulating concentration of glucose.

Early results showing the impact of reduced D-concentrations on living organisms were first published in 1993 [9]. Since then, numerous experimental and clinical observations have further suggested that deuterium depletion has an antimitotic effect in various tumor cells [9, 10]. This effect is, in part, due to the alteration of H^+^-ATPase and Na^+^/H^+^ antiport system [9]. Other studies have shown that deuterium-depleted water (DDW)-induced apoptosis in cancer cells, both in vitro and in vivo [10–12]. The inhibitory effect of DDW on the expression of proto-oncogenes such as c-Myc, Ha-ras and p53 has been documented, as well [13]. Complete or partial tumor regression has been established in mice xenografts with MDA-MB-231, MCF-7 human breast adenocarcinoma cell lines and PC-3 human prostate tumor cells, respectively. When laboratory animals were exposed to chemical carcinogenesis by 7,12-dimethyl-benzanthracene (DMBA), cytoplasmic myelocytomatosis oncogenes, c-Myc, Ha-ras, and p53 were up-regulated, while DDW, applied as drinking water, suppressed the expression of these oncogenes. DDW significantly inhibited proliferation of A549 human lung carcinoma cells in vitro, and H460 lung tumor xenografts in laboratory mice showed a 30% regression in growth. The anticancer effect of deuterium depletion has already been confirmed in a double-blind, randomized, 4-month-long, phase 2 clinical trial on prostate cancer, and the extended follow-up suggests that DDW delays the progression of the disease [14]. Retrospective clinical studies confirmed the anticancer effect of DDW as the consumption of DDW led to a several fold increase in the median survival time (MST) of patients with prostate, breast, lung and pancreatic cancer, respectively [14–17]. Deuterium-depleted water may be included as a non-toxic anticancer treatment modality for prevention and treatment. [18, 19].

At submolecular level, it has been shown that most of the extramitochondrial NADPH synthesis pathways are targeted by DDW. The terminal complex of mitochondrial electron transport chain (ETC) reduces molecular oxygen to deuterium-depleted metabolic water which affects gluconeogenesis as well as fatty acid oxidation. NADPH’s deuterium labeling depends on carbon-specific positional glucose deuterium enrichment, as well as deuterium enrichment of the cytoplasmic and mitochondrial water pools within cells [18, 19].Furthermore, switching from a ketogenic to high-sugar diet interferes with the deuterium-depleting action of mitochondria serving as a potential oncogenic initiator [18].

Multiple lines of evidence suggest that D_2_O inhibits insulin release from pancreatic islets by stabilizing the microtubular system of the β-cells or by inhibiting oxidative phosphorylation [20–22]. In addition, the intracellular H^+^-concentration plays a critical role in the glucose-induced, time-dependent potentiation of insulin release in pancreatic β-cells, as well [23]. Reduction in intracellular pH leads to the translocation of glucose transporters GLUT4 and GLUT1 to the sarcoplasma in the canine heart [24]. Based on these findings and the results confirming the key role of naturally occurring D in cell cycle regulation, it was logical to assume that deuterium depletion could interfere with glucose metabolism as well. Further evidence supporting this hypothesis was obtained from participants of clinical studies to verify the anticancer effect of deuterium depletion, where application of DDW in patients with cancer and diabetes mellitus (DM), resulted in significantly lower blood glucose levels [25].

Ingestion of 1.5 L DDW (104 ppm D) per day for 90 days was mostly beneficial by altering some parameters related to metabolic syndrome in patients with pre-diabetes or manifest diabetes mellitus. D content of the body had an impact on the physiological regulation in insulin-resistant patients. The fact that DDW simultaneously influenced insulin-, HDL- and glucose levels suggests that the concentration of D within the organism may have an important role in harmonizing metabolic processes. In summary, the results support the beneficial role of DDW in disorders of glucose metabolism [25]. In a phase 2 clinical study 30 volunteers with decreased glucose tolerance consumed DDW (104 ppm D) for 90 days. By the end of the trial the insulin concentration decreased in 50% of the volunteers, which correlated well with a decrease in glucose levels in the same participants. In addition, in 11 subjects glucose disposal was found to be improved significantly [25].

This study aimed to evaluate the effect of DDW on the whole-body glucose homeostasis in streptozotocin (STZ)-induced diabetic rat model as well as to elucidate the possible underlying mechanism. Here we show that DDW alone did not alter the glucose uptake of diabetic rats. However, DDW dose-dependently potentiates the effect of insulin, administered in a suboptimal dose, on glucose homeostasis. This effect, at least in part, may be attributed to the increased GLUT-4 protein translocation from the cytosol to the membrane of skeletal muscle cells.

## Materials and methods

### Animals

A total of 96 adult male Wistar rats (Charles River, Budapest, Hungary) weighing 180–200 g were used for the experiments. The animals were housed in hanging wire cages maintained at 21–22 °C, constant humidity, and 12 h light/12 h dark cycle (with lights on at 6.00 PM). Standard rat chow and water were given ad libitum.

The animal treatments were in compliance with guidelines established by the Animal Care Committee of Semmelweis University and the Hungarian Guide for the Care and Use of Laboratory Animals.

### Induction of diabetes

A period of 7 days was allowed for the animals to acclimatize before any experimental manipulations were performed. Prior to STZ treatment [26, 27], all animals were placed in metabolic cages for 24 h. Urine and 1 mL blood samples were collected to verify pretreatment values of the study parameters in all animals. Diabetes was induced in 12 h fasted rats with a single intraperitoneal injection of streptozotocin (STZ, Sigma, Budapest) 60 mg/bw-kgs, dissolved in 10 mmol sodium citrate buffer (pH:4.5) at a concentration of 90 mg/mL immediately before use. Age and weight matched control rats received an equal volume of citrate buffer only. After STZ injection, rat chow was given back immediately to prevent the development of hypoglycemia. All animals were kept under the same conditions and monitored closely for 2 weeks. To confirm the diabetic status, animals were placed again into metabolic cages for 24 h and urine and blood samples were collected. Only animals with polyuria, polydipsia, and plasma glucose concentrations above 15 mmol/L were considered diabetic and used in the study (Tables 1, 2).

**Table 1:** Effect of deuterium content of drinking water on the metabolic parameters of non-diabetic rats in a control group

DDW	Body weight	Chow	Water consumption	Diuresis	Glycoseuria
(ppm)	(g)	(g/100 g bw./day)	(ml/100 g bw./day)	(ml/100 g bw./day)	(mmol/100 g bw/day)
150	470.0 ± 32.7	6.2 ± 0.9	11.8 ± 1.9	5.3 ± 1.9	0.31 ± 0.1
	(8)				
25	449.0 ± 31.1	5.4 ± 0.4	10.0 ± 1.9	5.9 ± 1.7	0.33 ± 0.1
	(8)				

There were no statistically significant differences between the examined parameters

**Table 2:** Effect of deuterium content of drinking water on the metabolic parameters of STZ-treated rats without insulin administration

DDW	Body weight	Chow	Water consumption	Diuresis	Glycoseuria
(ppm)	(g)	(g/100 g.bw./day)	(ml/100 g.bw./day)	(ml/100 g.bw./day)	(mmol/100 g.bw./day)
150	243.0* ± 15.6	26.5* ± 9.5	156.3* ± 16.3	102.6* ± 24.0	120.6* ± 20.2
	(8)				
25	255.2* ± 29.6	24.8* ± 8.9	165.8* ± 21.6	105.1* ± 16.9	118.31* ± 23.5
	(8)				

There were no statistically significant differences between the examined parameters

Stars indicate significant differences (*p* < 0.001) compared to the corresponding values of the non-diabetic animals

### Experimental design A

To investigate the effect of DDW on metabolic changes occur in diabetes, diabetic and control rats were divided into two main groups: half of the animals were given DDW (25 ppm D), and the other half received normal tap water (150 ppm D), both provided ad libitum. To determine whether DDW exerts its effects, at least partially, by modifying the action of insulin, diabetic rats were further divided into subgroups according to insulin treatment. The control rats did not receive insulin treatment. We did not want to achieve euglycaemia by insulin treatment, but to prevent severe acute complications only. Therefore, two different doses of insulin (Huminsulin Lilly Normal 100 I.E./mL) were used: a low dose (1 IU/300 g bw./day) to prevent ketosis and a moderate dose (2 IU/300 g bw./day) to prevent severe hyperglycaemia.

The insulin was administered subcutaneously in equal portions twice daily (8.00 AM and 6.00 PM). Treatments started at the 2nd week after STZ injection and lasted for an additional 8 weeks.

### Experimental design B

To identify the most effective deuterium concentration, diabetic rats (n = 72) receiving a fixed low dose of insulin (1 IU/300 g bw./day) were treated with water containing different concentrations of deuterium (between 150 and 25 ppm D). According to this protocol, animals were divided into 9 subgroups based on the deuterium content of drinking water: Group25 (*n* = 8) 25 ppm D, Group75 (*n* = 8) 75 ppm D, Group105 (*n* = 8) 105 ppm D, Group125 (*n* = 8) 125 ppm D, Group130 (*n* = 8) 130 ppm D, Group135 (*n* = 8) 135 ppm D, Group140 (*n* = 8) 140 ppm D, Group145 (*n* = 8) 145 ppm D and Group150 (*n* = 8) 150 ppm deuterium-containing water, respectively. Treatments started at the 2nd week after STZ injection and lasted for 4 weeks.

Food intake, water consumption, and body weight were measured daily. Blood samples from the tail vein and 24 h urine samples were collected once a week. Blood samples were taken 4 h after insulin on 60 mg/mL EDTA (Sigma, Budapest, Hungary) as anticoagulant. The plasma samples were obtained by centrifugation at 3000×*g* for 15 min at 4 °C. 24 h urine samples were collected in tubes containing 200 µL of 10% boronic acid solution to prevent bacterial contamination. After measuring the volume, urine was centrifuged at 3000 × g for 10 min and the supernatant was used for analysis. All samples were stored at − 80 °C until elaboration.

At the end of the study, all animals were placed into metabolic cages individually for 24 h and exsanguinated under 60 mg/kg body weight pentobarbital (Nembutal, Abbott Labs, Chicago, IL, USA) anesthesia. After sacrificing the animals, soleus muscle was rapidly excised, washed in physiological saline solution, weighted, frozen in liquid nitrogen, and stored at − 80 °C until processing.

### Plasma glucose

Plasma Glucose was determined spectrophotometrically using reagent kits from Reanal Finechemical Co. (Budapest, Hungary).

### Insulin concentration

Insulin concentration was determined using an Insulin ELISA kit obtained from Agilent Dako (Santa Clara, CA). The plates were analyzed by a Biorad microplate reader.

### Plasma fructosamine concentration

Plasma fructosamine concentration was determined spectrophotometrically using the micro method developed by Oppel et al. [28], based upon the classical method of Johnson et al. [29], with minor modifications. In brief, fructosamine reagent was prepared by dissolving 50 mg nitroblue tetrazolium (NBT, from Sigma, Budapest, Hungary) in 244.6 mL carbonate buffer (pH:10.11). Standard was prepared from bovine serum albumin as described previously in detail [23]. Twenty µl of plasma or an adequate volume of standard solution was pipetted into the wells of a 96-well plate in three parallels, respectively. After the addition of 200 µl reagent into each well, the plates were profoundly shaken and then incubated at 37 °C for 10 min. The initial absorbance was read at 490 nm (A_1_). Following a subsequent incubation for 10 min, the absorbance was read again at 490 nm (*A*_2_). Fructosamine concentration (*C*_sample_) was calculated from Eq. 1, below *C*_standard_ represents the fructosamine concentration of the standard solution:

1 $${{(A2 - A1) \,{\rm{Sample}}} \over {(A2 - A1) \,{\rm{Standard}}}} \times C{ _{{\rm{Standard}}}} = C{ _{{\rm{Sample}}}}$$

### Glycosylated hemoglobin (HbA1c)

Glycosylated hemoglobin (HbA1c) was determined using an affinity chromatography method, previously described by Gould et al. [30]. In brief, 30 µl of blood samples (collected without anticoagulants) were hemolysated in 500 µL deionised water. Pierce™ Immobilized Boronic Acid Gel (Thermo Fischer Sci., Waltham, MA) was equilibrated in washing buffer (250 mmol ammonium acetate, 50 mmol magnesium chloride, 3 mmol sodium azide, pH = 8.5), and poured into columns (1 mL). After further washing of the columns with washing buffer (10 mL), 100 µL of the hemolysate were transferred to the top of the columns and allowed to soak in. Unbound hemoglobins were eluted by passing 8 mL of washing buffer through the column. Bound (glycosylated) hemoglobin was then eluted with 3 mL of elution buffer (200 mmol sorbitol, 50 mmol EDTA, 3 mmol sodium azide, 100 mmol Tris, pH = 8.5). The unbound fraction, containing most of the hemoglobin was diluted to 15 mL with washing buffer. The absorbance of each fraction was measured at 405 nm and the amount of hemoglobin bound (glycosylated) was calculated as a percentage of the total.

### Isolation of the membrane fraction of soleus muscle

Isolation of the membrane fraction of soleus muscle was performed according to Villanueva-Peñacarrillo ML et al. [31] supplemented by Lise Coderre et al.—ultracentrifugal separation process [32]. Briefly, soleus muscles from each rat hind limb were removed and trimmed of connective tissue, fat and nerves. One gram of tissue was minced well and homogenized in 10 ml of lysis buffer (20 mM Tris–HCl, 5 mM EDTA, 10 mM EGTA, 2 mM DTT, 1 mM Na_3_VO_4_, 25 μg/mL PMSF, 2.5 μg/mL Leupeptin, 2.5 μg/mL Aprotinin, 625 μM sodium-pyrophosphate, 1 mM β-glycerophosphate, 0,1% Triton) by a Polytron homogenizer for 3 × 15 s on ice. The homogenate was centrifuged at 2500×*g* for 10 min at 4 °C, and the pellet was discarded. The supernatant was then centrifuged at 100 000×*g* for 60 min in a Beckman SW55 rotor. The pellet was resuspended in 0.5 mL lysis buffer containing 38% sucrose and 1.5 mL each of 25, 30 and 35% wt/wt sucrose solution were layered on the top and centrifuged at 50.000×*g* for 16 h. Membrane fraction from the interphase 25/30% was collected, diluted with washing buffer (250 mM sucrose, 1 M NaCl, 10 mM Tris–HCl, 10 mM EDTA, 3.3 μg/mL PMSF, 1 μg/ml Aprotinin) and recovered by centrifugation at 100,000×*g* for 60 min. The pellet was finally resuspended and homogenized in washing buffer, and the total membrane protein content was measured by the Bradford method. Following protein assay, samples were stored at − 80 °C and used within four weeks for GLUT4 assay. Membranes collected from the interphase 25/30 are considered the plasma membrane fraction, since they show, by convention, enrichment in plasma membrane markers [33].

### Western blot analyses

The samples were prepared in 2 × Laemmli buffer containing 100 mmol dithiothreitol and boiled in a water bath for 15 min. Protein (50 μg) was separated on an SDS-PAGE (10%) gel followed by a wet transfer to a nitrocellulose membrane for 90 min. Total protein was visualized by Ponceau staining, and GLUT4 blots were normalized to the 45 kDa band. The membranes were blocked for 1 h at room temperature in 10% (wt/vol) nonfat dried milk in Tris-buffered saline (TBS) with 0.1% Tween 20 (TBST) and then incubated overnight with antibodies against the GLUT4 glucose transporter, purchased from Calbiochem-Sigma (Budapest, Hungary) and diluted (1:3000) in 1% bovine serum albumin in TBST. Blots were incubated with an HRP-conjugated secondary antibody in TBST for 1 h at room temperature and visualized by Enhanced Chemiluminescence (ECL) (BioRad, Budapest, Hungary). The ODs of bands were determined by densitometry. All chemicals not mentioned otherwise were purchased from Sigma (Budapest, Hungary).

### Statistical analyses

The results are presented as the mean ± SEM of n observations. The data were subjected to either one-way repeated measures analysis of variance (ANOVA) or post hoc Student–Newman–Keuls test or were evaluated by Student’s *t* test. Differences of *p* < 0.05 were considered significant.

## Results

### DDW as drinking water reduced plasma glucose concentration and improved the metabolic parameters in diabetic rats

**Fig. 1:**
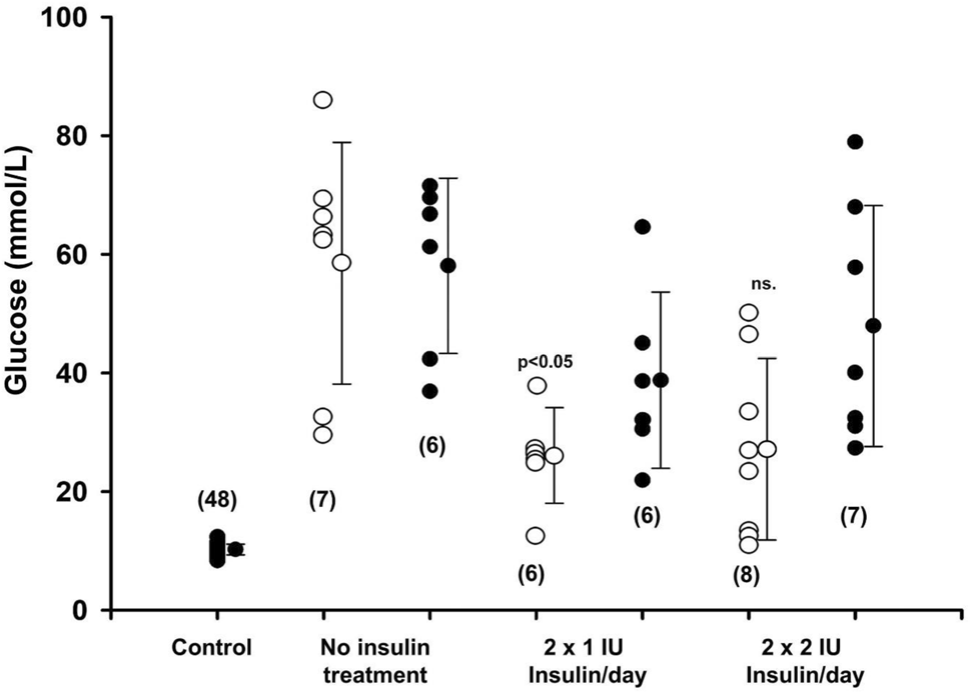
The effect of DDW (25 ppm) on the serum glucose level of diabetic rats treated with low (twice 1 IU/300 g bw/day) and moderate dose (twice 2 IU/300 g bw/day) of insulin at the end of the fourth week of treatment. Administration of insulin in a low dose significantly (*p* < 0.05) reduced the serum glucose level in DDW-treated animals only. Open and closed circles represent the data obtained from animals receiving either DDW (25 ppm) or tap water, respectively. Numbers in brackets show the number of animals in each group

To evaluate the effect of DDW on the glucose metabolism in streptozotocin (STZ)-induced diabetic rat model, first we aimed to test the 25 ppm D-concentration, which was the lowest D-concentration of DDW available. In animals without any insulin treatment, DDW alone did not affect blood glucose concentration. However, in animals that received both insulin and DDW, the glucose concentration was lower compared to the animals receiving tap water only, and the difference was significant when a low dose of (2 × 1 IU/300×*g* bw./day) insulin was used (Fig. 1). These results indicate that deuterium depletion, in the presence of insulin, can affect blood glucose levels in diabetic animals.

To evaluate the changes of metabolic parameters in non-diabetic rats receiving drinking water with 150 ppm or 25 ppm D content for four weeks were individually placed in metabolic cages for 24 h and the amount of fluid and food consumed was measured and the volume of urine excreted and its glucose content were determined.

To evaluate the changes of metabolic parameters in STZ-treated diabetic rats receiving drinking water with 150 ppm or 25 ppm D content for four weeks were individually placed in metabolic cages for 12 h, without insulin administration, and the amount of fluid and food consumption, the volume of urine excretion and its glucose content were measured, respectively.

In conclusion, the D content of the drinking water did not significantly affect the parameters studied in either non-diabetic or STZ-treated animals without insulin administration.

**Fig. 2:**
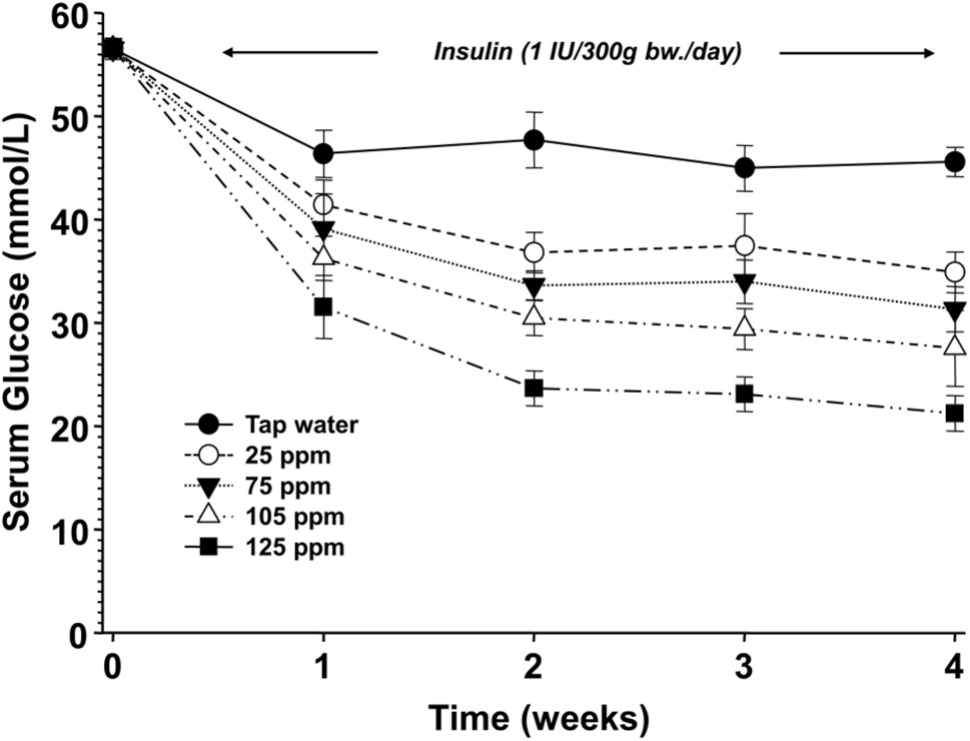
The effect of deuterium content of drinking water on the serum glucose concentration in diabetic rats treated with insulin (twice 1 IU/300 g bw./day)

Next, to determine the most effective D-concentration, DDW with 25, 75, 105, and 125 ppm D-concentrations was tested along with the application of a low insulin dose only. Surprisingly, we found that the 125 ppm value, which was closer to the natural D level, was the most effective D-concentration in reducing blood glucose levels in diabetic animals. (Fig. 2).

The milder decrease of deuterium content of drinking water exerted the greatest effect on serum glucose level. (All data points were significantly (*p* < 0.01) lower compared to the animals receiving tap water). The inset shows the values for the symbols.

To test the dose-dependent effect of deuterium content of drinking water on the metabolic parameters STZ-treated rats receiving various concentrations of DDW as drinking water for four weeks were individually placed in metabolic cages for 12 h, after receiving their insulin injections (1 IU insulin/300 g body weight). The amount of fluid and food consumed was measured, and the volume of urine excreted and its glucose content were determined. (Table 3.)

**Table 3:** Effect of deuterium content of drinking water on the metabolic parameters of diabetic rats treated with insulin (twice 1 IU/300 g bw./day)

DDW	Body weight	Chow	Water consumption	Diuresis	Glycosuria
(ppm)	(g)	(g/100 g.bw./day)	(ml/100 g.bw./day)	(ml/100 g.bw./day)	(mmol/100 g.bw./day)
150	258.3 ± 33.3	24.2 ± 3.43	145.2 ± 18.2	83.3 ± 17.5	119.7 ± 16.8
	(8)				
125	350.0 ± 36.7	10.9 ± 1.4^++^	42.4 ± 4.5^++^	19.3 ± 2.9^++^	47.1 ± 9.8^++^
	(8)				
105	323.2 ± 36.8	16.3 ± 3.4^+^	82.1 ± 16.9^+^	35.6 ± 20.6^+^	63.7 ± 20.9^+^
	(7)				
75	315.1 ± 24.0	17.4 ± 2.4	87.8 ± 19.1	50.7 ± 16.0	88.5 ± 26.8
	(8)				
25	306.4 ± 34.7	19.2 ± 2.7	95.9 ± 22.4	71.0 ± 21.1	93.6 ± 16.5
	(8)				

**Table ****3****.** Effect of deuterium content of drinking water on the metabolic parameters of diabetic rats treated with insulin (twice 1 IU/300 g bw/day).

All evaluated parameters were significantly different (*p* < 0.001) from the corresponding values of the non-diabetic animals. + (*p* < 0.05), + + (*p* < 0.01) are significant differences compared to the corresponding values of the 150-ppm-DDW-receiving animals.

**Fig. 3:**
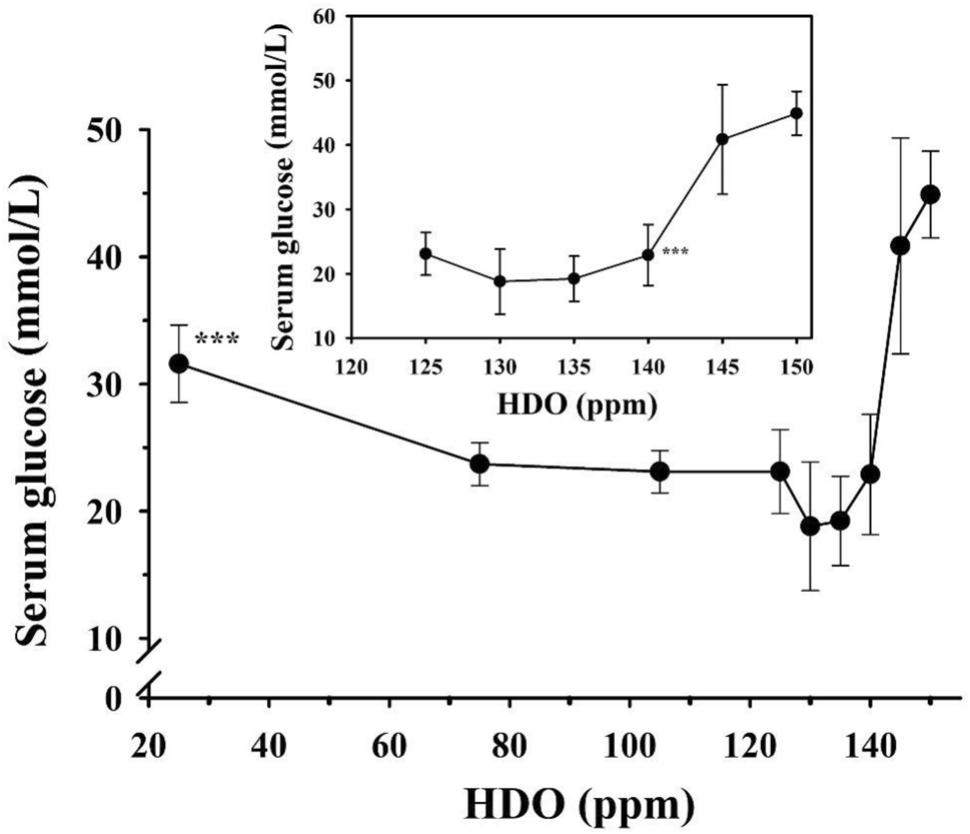
The effect of deuterium content of drinking water on the blood glucose level in diabetic rats treated with insulin (twice 1 IU/300 g bw/day)

**Fig. 4:**
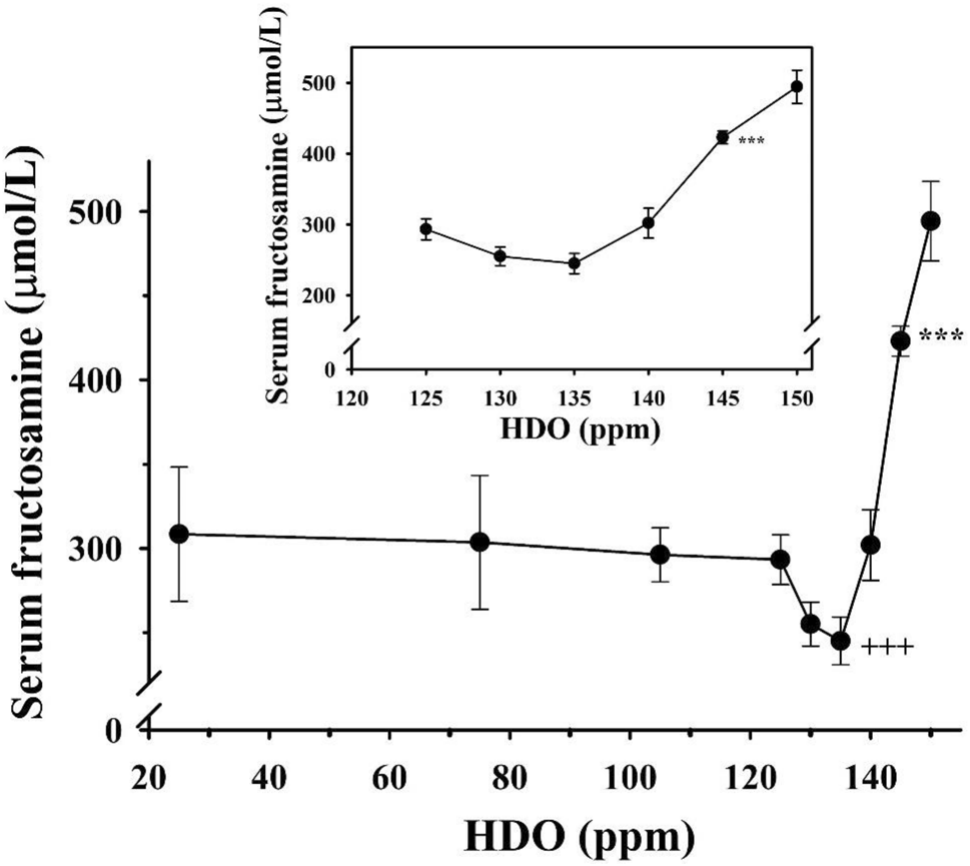
The effect of deuterium content of drinking water on the fructosamine level in diabetic rats treated with insulin (twice 1 IU/300 g bw/day)

**Fig. 5:**
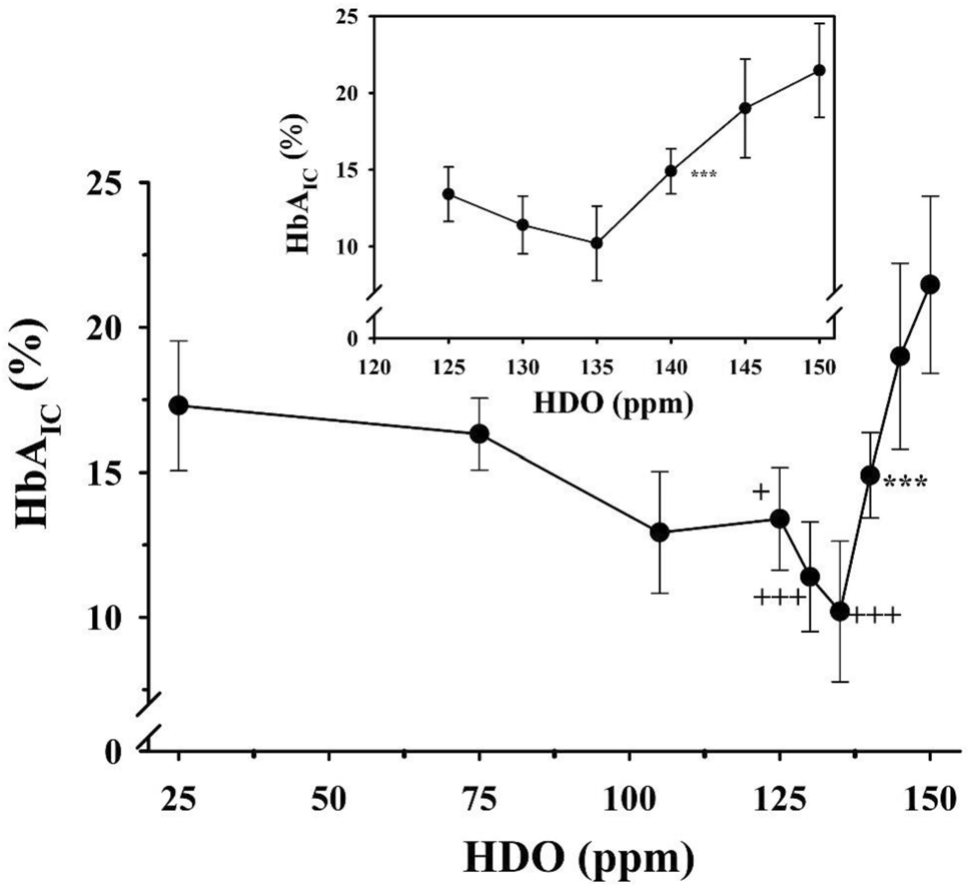
The effect of deuterium content of drinking water on the HbA1c level in diabetic rats treated with insulin (twice 1 IU/300 g bw./day)

Figures 3–5 show the summary of three experiments in which the following D-concentrations were applied: 25, 75, 105, 125, 130, 135, 140, 145 ppm and 150 ppm in the control group. Low dose of insulin was used along with all D-concentrations.

The results revealed that the most effective D-concentrations were in the range of 125–140 ppm (Fig. 3), which was further confirmed by measurements of fructosamine and HbA1c concentrations (Figs. 4, 5).

The inset graphs show the magnified part of the curve between the deuterium content of 125 ppm and 150 ppm, respectively. Animals receiving drinking water containing below 140 ppm deuterium had significantly (***p < 0.01) lower blood glucose levels than those that had access to tap water.

The inset graphs show the magnified part of the curve between the deuterium content of 125 ppm and 150 ppm, respectively. Animals receiving less than 145 ppm deuterium-containing drinking water had significantly (***p < 0.01) lower fructosamine levels than those which had access to tap water. Animals receiving drinking water-containing 135 ppm deuterium had the lowest fructosamine levels among the DDW-treated animals. Three pluses, (++ + *p* < 0.01) versus the value for rats receiving drinking water-containing 25 ppm deuterium.

The inset graphs show the magnified part of the curve in the range of the deuterium content between 125 and 150 ppm. Animals receiving drinking water below140 ppm deuterium content had significantly (***p < 0.01) lower HbA1c values than those which had access to tap water (150 ppm). Furthermore, symbols indicate the significance level of data compared to animals receiving 25 ppm deuterium-containing drinking water. (+ meaning *p* < 0.05; + + *p* < 0.025; + + + *p* < 0.01).

### DDW did not influence the half-life of insulin in blood

**Fig. 6:**
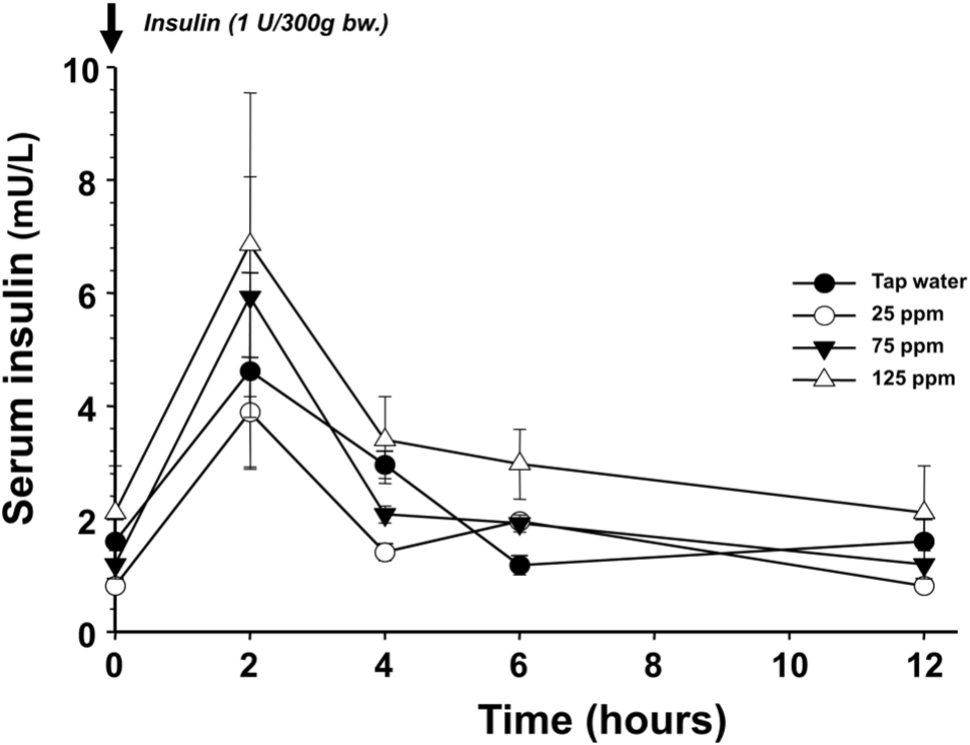
The elimination of a single dose (1 IU/300 g bw.) of human insulin has not been altered by the concentration of deuterium in the drinking water in STZ-treated rats

The first experiment revealed that the presence of insulin is essential for DDW to be able to reduce blood glucose concentration. To exclude the possibility that deuterium depletion may stabilize the insulin in the plasma which results in lower blood glucose levels, the insulin concentration of the blood plasma was determined after the administration of DDW. The insulin concentration was the highest two hours after administration and there was a gradual decrease for 10 h until the next administration, but the data did not support the hypothesis that DDW influences insulin stability (Fig. 6).

The symbols represent different deuterium contents in the drinking water: tap water (closed circle), 25 ppm (open circle), 75 ppm (closed inverted triangle), 125 ppm (open triangle), respectively.

**Fig. 7:**
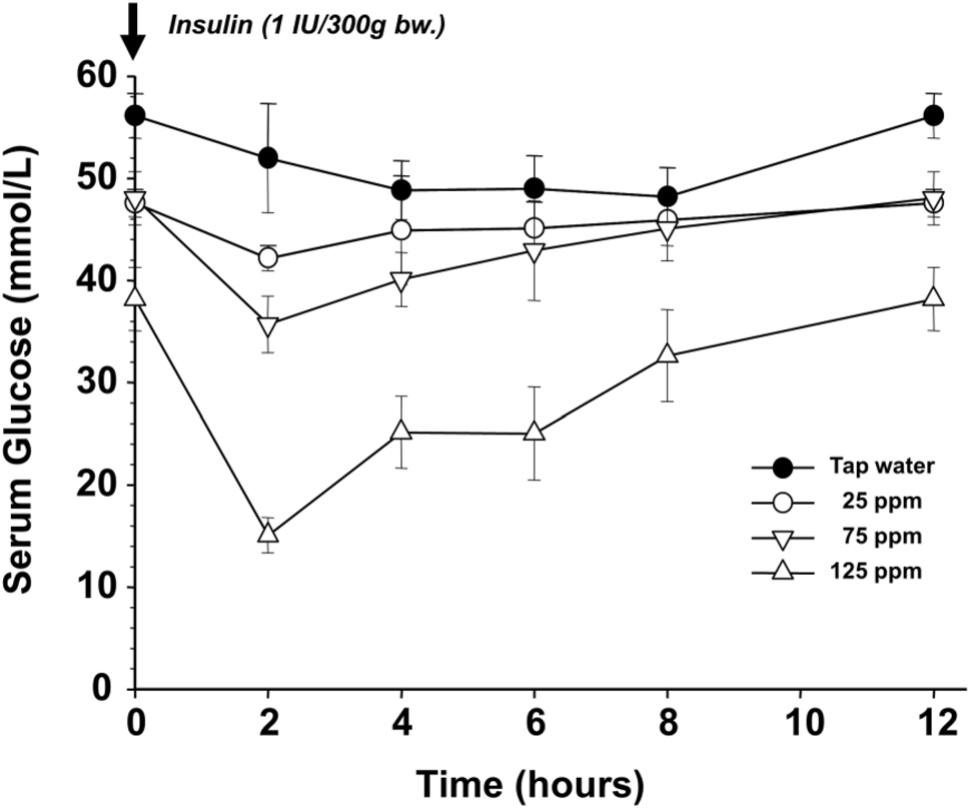
Changes of serum glucose concentration after the administration of 1 IU/300 g bw. insulin to STZ-treated animals receiving various deuterium concentration categories of drinking water

The essential role of insulin in the mechanism influenced by the lowering D-concentration was also confirmed by following the correlation between insulin and glucose concentration after every two hours of administration. The data show that the blood glucose concentration was the lowest two hours after the administration, when the insulin level was the highest. The glucose concentration was 52 mmol/L in the control group, but 43 mmol/L, 36 mmol/L, and 15 mmol/L in correlation with the D-concentrations of 25, 75 or 125 ppm, respectively (Fig. 7).

The symbols represent different deuterium contents of the drinking water: tap water (closed circle), 25 ppm (open circle), 75 ppm (closed inverted triangle), 125 ppm (open triangle), respectively. All data points of 125 ppm deuterium are significantly (*p* < 0.05) lower than the values obtained by other treatments.

### DDW dose-dependently potentiates the effect of insulin, in part, due to the increased GLUT4 protein translocation from the cytoplasm to the membrane

**Fig. 8:**
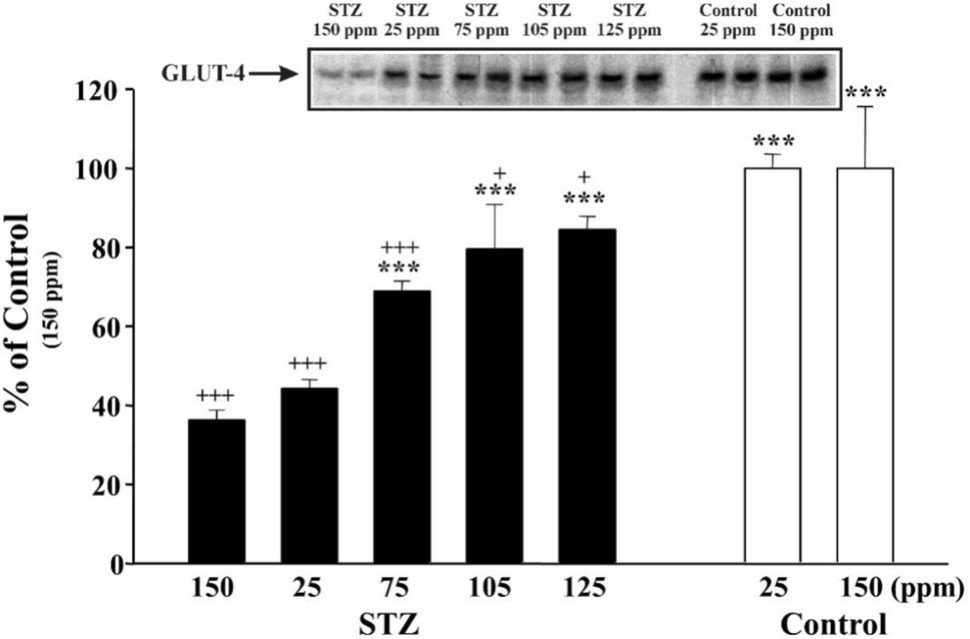
The effect of the deuterium content of drinking water on insulin-induced translocation of GLUT4 to the membrane in the soleus muscle

As glucose is cleared from the bloodstream by a family of facilitative transporters (GLUTs) and the GLUT4 isoform is the major insulin-responsive transporter, we planned to measure the amount of GLUT4 in the membrane fraction of the soleus muscle. Figure 8 shows that in non-diabetic rats the GLUT4 level was high and remained the same independently of D-concentration in the drinking water (25 ppm or 150 ppm).

For the experimental protocol, see the Materials section. Inset shows a representative immunoblot of insulin-promoted GLUT4 translocation to the membrane fraction. Bars indicate the mean values of the densitometry evaluation of the GLUT4 protein corresponding spots on the immunoblots and expressed as a percentage of the value of the tap water given to control group (non-diabetic) animals. Three asterisks indicate *p* < 0.01 versus STZ-treated and tap-water-receiving animals. Three pluses indicate *p* < 0.01, one plus *p* < 0.05, versus control, non-diabetic animals.

In diabetic rats, we found a strong correlation between the amount of GLUT4 in the membrane fraction and the blood glucose concentration. As the blood glucose concentration was the highest in the control group, the lowest GLUT4 level was detected in the membrane, but at the same time the 125 ppm DDW resulted in the lowest glucose concentration. This can be explained with the highest GLUT4 level being in the membrane.

## Discussion

There has been increasing evidence that naturally occurring deuterium has a central role in living organisms since the first paper was published [9]. In the early 90s, research primarily focused on the anticancer effect of DDW [9–19], but for today it is clear that D and more probably the changing D/H ratio has an exceptional role in the regulation of biochemical-, genetic- and physiological processes [13, 25, 34, 35]. In this study, we proved that the changes in D-concentrations in water may potentiate the insulin-regulated membrane trafficking by recruiting membrane vesicles containing the GLUT4 glucose transporters from the interior of cells to the cell surface. One of the most striking results was that it was not the lowest D-concentration (25 ppm) that exerted the most significant stimulus on the insulin signal transduction system, but a narrow subnatural concentration range between 125 and 140 ppm. These findings suggest that the results cannot be attributed only to the kinetic isotope effect, because in this case the lowest D-concentration would exert the strongest effect, and the hitherto unknown submolecular regulatory system is very sensitive to changes in the D/H ratio in this range. Previous work [9] suggested that a Na^+^/H^+^ antiport activation may lead to an increased D/H ratio because the transport system prefers to eliminate the lighter isotope (H^+^), which may serve as a key signal to promote cell proliferation [9]. Recent results suggest that properly working mitochondria can keep the D/H ratio at a lower level, because lipid oxidation provides deuterium-depleted metabolic water [17–19]. We also suggest that the D-concentration varies in the different nutrients depending on the place of cultivation, country of origin, the biochemical pathways for photosynthetic CO_2_-fixation in plants [36, 37], and the ratio of the main organic compounds (carbohydrates, proteins, lipids) also influences cellular processes and has a major impact on metabolism.

Based on our experimental data, deuterium-depleted water can offer clinical benefits in the treatment of patients with metabolic syndrome by increasing insulin sensitivity. The results presented here serve as novel evidence that the naturally occurring deuterium has an important role in living organisms.

Further research is needed to explore whether a similar mechanism could be responsible for the beneficial effect of DDW on the parameters of glucose metabolism in human subjects, however this is beyond the scope of the current study. Nevertheless, deuterium depletion can potentially offer an effective and innocuous tool for treating not only malignant but also metabolic diseases.

## Data Availability

All data generated or analysed during this study are included in this published article.
